# Gene Mutation Analysis in 253 Chinese Children with Unexplained Epilepsy and Intellectual/Developmental Disabilities

**DOI:** 10.1371/journal.pone.0141782

**Published:** 2015-11-06

**Authors:** Yujia Zhang, Weijing Kong, Yang Gao, Xiaoyan Liu, Kai Gao, Han Xie, Ye Wu, Yuehua Zhang, Jingmin Wang, Feng Gao, Xiru Wu, Yuwu Jiang

**Affiliations:** 1 Department of Pediatrics and Pediatric Epilepsy Center, Peking University First Hospital, Beijing, China; 2 Center of Epilepsy, Beijing Institute for Brain Disorders, Beijing, China; 3 Department of Neurosurgery, the Second Hospital of Dalian Medical University, Dalian, China; 4 The Children’s Hospital, Zhejiang University School of Medicine, Zhejiang, China; University of Texas Health Science Center, UNITED STATES

## Abstract

**Objective:**

Epilepsy and intellectual/developmental disabilities (ID/DD) have a high rate of co-occurrence. Here, we investigated gene mutations in Chinese children with unexplained epilepsy and ID/DD.

**Methods:**

We used targeted next-generation sequencing to detect mutations within 300 genes related to epilepsy and ID/DD in 253 Chinese children with unexplained epilepsy and ID/DD. A series of filtering criteria was used to find the possible pathogenic variations. Validation and parental origin analyses were performed by Sanger sequencing. We reviewed the phenotypes of patients with each mutated gene.

**Results:**

We identified 32 novel and 16 reported mutations within 24 genes in 46 patients. The detection rate was 18% (46/253) in the whole group and 26% (17/65) in the early-onset (before three months after birth) epilepsy group. To our knowledge, we are the first to report *KCNAB1* is a disease-causing gene of epilepsy by identifying a novel *de novo* mutation (c.1062dupCA p.Leu355HisfsTer5) within this gene in one patient with early infantile epileptic encephalopathy (EIEE). Patients with an *SCN1A* mutation accounted for the largest proportion, 17% (8/46). A total of 38% (9/24) of the mutated genes re-occurred at least 2 times and 63% (15/24) occurred only one time. Ion channel genes are the most common (8/24) and genes related to synapse are the next most common to occur (5/24).

**Significance:**

We have established genetic diagnosis for 46 patients of our cohort. Early-onset epilepsy had the highest detection rate. *KCNAB1* mutation was first identified in EIEE patient. We expanded the phenotype and mutation spectrum of the genes we identified. The mutated genes in this cohort are mostly isolated. This suggests that epilepsy and ID/DD phenotypes occur as a consequence of brain dysfunction caused by a highly diverse population of mutated genes. Ion channel genes and genes related to synapse were more common mutated in this patient cohort.

## Introduction

Epilepsy and intellectual/developmental disabilities (ID/DD) are both common pediatric neurological disorders. ID/DD is one of the main comorbidities of epilepsy [1] with about a quarter of epileptic children having ID/DD. The prevalence is much higher in children presenting with early-onset (before three months after birth) epilepsy [2–4]. Although frequent and refractory seizures may cause cognitive and motor regression, common pathophysiological mechanisms may be responsible for the high rate of co-occurrence of epilepsy and ID/DD [5].

The etiologies of these two disorders are complex and diverse with the majority being unknown. Genetic factors play a major role in the etiologies of epilepsy and ID/DD, especially in pediatric patients, who are highly heterogeneous. Defects in many genes have been reported as shared underlying mechanisms of epilepsy and ID/DD [6–9]. These genes are involved in different pathways. Ion channel genes, which are particularly relevant to epilepsy, account for a significant proportion [10]. However, the phenotypes related to these genes are difficult to differentiate clinically, and the detection of mutations in suspected genes is always a challenge. Seizures were always intractable and indicated a poor prognosis when co-occurring with ID/DD. Therefore, knowing the genetic background and pathogenesis of epilepsy and ID/DD is valuable not only for diagnosis and prognosis, but also for genetic counseling and treatment.

Many genes related to epilepsy and ID/DD have been reported. However, for individual patients with non-syndromic epilepsy, it has been difficult to isolate the causative gene mutations from a large number of possible candidate genes using conventional Sanger sequencing. The rapidly developing targeted next-generation sequencing (NGS) has been proved to be a fast, economic and accurate approach for screening gene mutations in disorders with both genetic and phenotypic heterogeneity, including epilepsy and ID/DD [11]. In this study, we used targeted NGS to investigate 300 candidate genes related to epilepsy and ID/DD in 253 Chinese children with unexplained epilepsy and ID/DD. We aim to make genetic diagnosis for these patients and find clues to help us explain the common genetic background of epilepsy and ID/DD.

## Methods

### Ethics statement

Written informed consent was obtained from the parents of all the patients. This study was approved by the Institutional Review Boards of Peking University First Hospital. All data of this study were analyzed anonymously.

### Patients

A total of 253 Chinese children with unexplained epilepsy and ID/DD were recruited from the Department of Pediatrics, Peking University First Hospital from 2006 to 2014. All patients were clinically diagnosed as having epilepsy and ID/DD of unknown origin, including 65 patients diagnosed as early-onset (before three months after birth) epilepsy. Nevertheless, it was strongly suspected that the etiology of these patients’ diseases was genetic, owing to the following evidences: (1) no definite perinatal brain injury; (2) no hypoxia, ischemia, infection of the central nervous system or cranial trauma; (3) no evidence of typical inherited metabolic disorders or specific neurodegenerative disorders based on clinical features, neuroimaging or blood/urinary metabolic diseases screening; (4) normal routine karyotyping and (5) the detection of chromosome sub-telomeric rearrangements with multiplex ligation-dependent probe amplification (MLPA) showing no abnormalities [12]. 253 cases of our cohort included cases from 246 trios, of which father or mother of four trios (1649, 5165, 5168, and 5237) had epilepsy history, and parents of all the other 242 trios had no epilepsy and any related history. Other seven cases were probands of seven families with more than one patient, of which six families (3604, 5750, 6047, 6364, 6526, and 6636) consisted of two affected children and their unaffected parents, and one family (5240) with five affected patients (proband, father, uncle, aunt and grandfather) accorded with autosomal dominant inheritance. All genomic DNA used in experiments were extracted from peripheral leukocytes.

### Targeted next-generation sequencing

In accordance with the literatures searched within online databases, a total of 300 candidate genes associated with epilepsy and ID/DD were selected as the genes of interest. We used a custom-designed gene panel, synthesized using the Agilent Sure-Select Target Enrichment technique (Zhongguancun Huakang Gene Institute, China), to capture the coding regions from the 300 genes, including their exons and exon-intron boundaries (1.285M bp in total). The following NGS was performed on an Illumina GAIIx platform (Illumina, San Diego, CA, U.S.A.) using paired-end sequencing of 110 bp. Bioinformatics analysis of the raw data included the following steps: (1) image analysis using RTA software (real-time analysis, Illumina); (2) base calling using CASAVA software v1.8.2 (Illumina); (3) filtered out duplicate and low base quality score reads using the Genome Analysis Tool kit (GATK); (4) aligned clean paired-end reads to the human reference genome build hg19 using BWA software (Pittsburgh Supercomputing Center, Pittsburgh, PA, U.S.A.); (5) identified insertion-deletions (indels) and single-nucleotide polymorphisms (SNPs) using the GATK and annotated using ANNOVAR; (6) performed in silico pathogenicity prediction of novel missense variations using Polyphen2 [12, 13].

The sequencing depth was more than 5X (range of 5X-185X; average of 136X), and the mean coverage was 98.56%. On average, 423 variations within the 300 genes were found in each patient. We then formulated the following filtering criteria to determine every possible pathogenic variation from the large amount of initial variations: (1) insertion/deletion variations; (2) premature/delayed termination codon variations; (3) splice site variations including substitution at nucleotide +1/-1 of intron; (4) missense variations predicted by polyphen2 as probably/possibly damaged or benign. The variation meeting any one of the above criteria was considered to be a candidate for pathogenic variations and was selected for further analysis [12].

According to the HGMD Professional database, the 1000 Genomes Browser, PubMed and the UCSC database, we marked the reported pathogenic mutations and excluded known polymorphisms. Finally, on average 17 possible pathogenic variations were identified in each patient (range 5–27). We chose variations which to validate according to the known inheritance pattern of the involved genes. Heterozygous variations of genes with autosomal or X-linked dominant heredity, homozygous or compound heterozygous variations of genes with autosomal recessive heredity, and hemizygous variations of genes with X-linked recessive heredity were regarded as likely causative variations. We performed validation and parental origin analyses for these variations by conventional Sanger sequencing, and confirmed causative mutations according to parental origin of the variations and clinical features of the patients [12].

### Protein structure modelling

The homology modelling server SWISS-MODEL [14–16] was used to predict the tertiary structure of *KCNAB1* protein. We chose the protein crystal structure 3EAU [17] of *KCNAB2* from Protein Data Bank (PDB) [18] as the template. For the homology of *KCNAB1* and *KCNAB2*, the structure from 89 to 413 amino acids of *KCNAB1* protein (UniProtKB ID: Q14722) were predicted.

## Results

We identified causative mutations within 24 genes in 46 patients of our cohort, including two likely pathogenic mutated genes in two patients. The total detection rate of our study was 18% (46/253) in the whole group and 26% (17/65) in the early-onset epilepsy group. The detected mutations included 32 novel and 16 reported mutations. Nineteen of the mutations were severe, including eight premature/delayed termination codon mutations, ten insertion/deletion mutations and one splicing site mutation. The remaining 29 mutations were non-synonymous, including 27 mutations predicted to be “probably damaging” and two mutations (*AFF2* p.Gly547Asp and *RELN* p.Val3426Ile) that were predicted to be “possibly damaging” by Polyphen2. In two patients (5240 and 6189) with distinct epilepsy and ID/DD phenotypes, two mutated genes were regarded as likely pathogenic due to the unmatched phenotype or the unavailability of segregation analysis in the family. An overview of the clinical features of patients and their mutations is described in Table 1.

**Table 1:** Overview of clinical features of the patients and the mutations.

Gene	Case	Sex	Study age (y)	Seizures (onset age)	ID/DD	Nucleotide substitution	Amino acid substitution	Parental origin	Novel/ reported	Final diagnosis
*SCN1A*	152	M	8	GTCS, FS (6 m)	Severe	c.4547C>A	p.Ser1516Ter	De novo	Reported	DS
	2038	M	10	GTCS, PS (4 m)	Severe	c.2134C>T	p.Arg712Ter	De novo	Reported	DS
	5791	M	3	PS, GTCS (3 m)	Severe	c.4942C>T	p.Arg1648Cys	De novo	Reported	DS
	6047	M	3	PS, FS, Myoclonus, Absences (8 m)	Severe	c.2589+3A>T	-	De novo	Reported	DS
	6207	F	15	PS. GTCS, FS (3 m)	Severe	c.3733C>T	p.Arg1245Ter	De novo	Reported	DS
	6222	F	0.5	PS, SE (16 d)	Severe	c.659T>A	p.Val220Asp	De novo	Novel	MMPSI
	6300	F	3	PS, Myoclonus, GTCS, SE, FS (6 m)	Severe	c.3372delT	p.Phe1124LeufsTer4	De novo	Novel	DS
	6492	M	4	PS, FS (7 m)	Severe	c.2488G>T	p.Glu830Ter	De novo	Novel	DS
*SCN8A*	3129*	M	9.5	GTCS, FS (11 m)	Severe	c.2668G>A	p.Ala890Thr	De novo	Novel	
	5487*	M	3.5	GS (6 m)	Severe	c.4850G>A	p.Arg1617Gln	De novo	Reported	EE
	6219*	F	1.5	PS, GTCS, Myoclonus, IS (3 d)	Severe	c.1221G>C	p.Leu407Phe	De novo	Novel	EIEE
	6325*	F	1	PS, GTCS (2.5 m)	Moderate to severe	c.2549G>A	p.Arg850Gln	De novo	Novel	EIEE
	6908*	M	1.5	GTCS (4 m)	Moderate to severe	c.4787C>G	p.Ser1596Cys	De novo	Novel	EE
	YL	F	3	PS (6 m)	Severe	c.4935G>A	p.Met1645Ile	De novo	Novel	
*KCNQ2*	597	M	3	GS (3 d)	Severe	c.365C>T	p.Ser122Leu	De novo	Reported	EIEE
	2202	M	2	PS, GTCS, FS (3 d)	Severe	c.956A>C	p.Lys319Thr	De novo	Novel	EIEE
	2312	M	1.5	PS, FS (4 h)	Severe	c.830C>T	p.Thr277Ile	De novo	Novel	EIEE
	5630	M	1	PS, IS (3 d)	Severe	c.1655A>C	p.Lys552Thr	De novo	Reported	EIEE
*IQSEC2*	3481	M	9	PS (3 y 6 m)	Severe	c.88_90delATC	p.Ile30del	De novo	Novel	
	3604	M	7	PS, FS (6 m)	Severe	c.1049C>A	p.Ala350Asp	Mother	Novel	
	5292	M	2	PS, IS, Myoclonus (1 y 4 m)	Severe	c.2846_2852delCCCAGGT	p.Ser949CysfsTer7	De novo	Novel	
*CDKL5*	1613	F	1	PS (40 d)	Severe	c.2314delA	p.Lys772ArgfsTer12	De novo	Novel	EIEE
	5057	F	1.5	PS (1 m)	Severe	c.528G>A	p.Trp176Ter	De novo	Novel	EIEE
*DYRK1A*	2091	M	7	GTCS, FS, SE (1 y 6 m)	Severe	c.859G>T	p.Asp287Tyr	De novo	Novel	
	2959	F	3	GS (2 y)	Severe	c.946C>T	p.Gln316Ter	De novo	Novel	
*GABRB3*	1538	M	1	PS, GTCS (6 m)	Severe	c.914C>T	p.Ala305Val	De novo	Novel	
	SYH	M	1.5	PS (9 m)	Severe	c.509T>G	p.Leu170Arg	De novo	Novel	
*PCDH19*	6526	F	9	PS (5 m)	Severe	c.1091delC	p.Pro364ArgfsTer4	Father	Reported	
	LXX	F	3	PS, FS (9 m)	Moderate	c.370G>A	p.Asp124Asn	Father	Novel	
*STXBP1*	527	M	2	IS (7 d)	Severe	c.568C>T	p.Arg190Trp	De novo	Reported	EIEE
	MYS	M	2	IS (3 d)	Severe	c.568C>T	p.Arg190Trp	De novo	Reported	EIEE
*AFF2*	6636	M	11	PS (6 y)	Moderate	c.1640G>A	p.Gly547Asp	Mother	Novel	
*ALDH7A1*	5921	M	4	PS (3 m)	Moderate to severe	c.1553G>C c.1061A>G	p.Arg518Thr p.Tyr354Cys	FatherMother	Novel Reported	PDE
*ATP1A2*	5871	M	1.5	PS, FS (1 m)	Mild	c.2563G>A	p.Gly855Arg	De novo	Reported	
*CASK*	2584	F	6	IS (1 y)	Severe	c.2141delC	p.Ala714GlufsTer13	De novo	Novel	MICPCH
*FOXG1*	2539	M	2	GTCS, PS, Laugh attack (6 m)	Severe	c.738C>A	p.Tyr246Ter	De novo	Novel	
*GRIN2A*	6245	F	6	PS, ESES (5 y)	Severe	c.2191G>A	p.Asp731Asn	De novo	Reported	
*GRIN2B*	1503	M	2	PS, IS, Myoclonus, Tonic, Startle (6 m)	Severe	c.1985A>C	p.Gln662Pro	De novo	Novel	
*KCNAB1*	HY	F	4	PS, GTCS (10 d)	Severe	c.1062dupCA	p.Leu355HisfsTer5	De novo	Novel	EIEE
*PRRT2*	5240	F	1	GS, IS (3 m)	Severe	c.649dupC	p.Arg217ProfsTer8	Father	Reported	
*RELN*	6235	F	7	Myoclonus (3 y)	Severe	c.10276G>A c.2252A>C	p.Val3426Ile p.Lys751Thr	Father Mother	Novel Novel	
*SHANK3*	ZXT	M	3	IS (1 y 10 m)	Severe	c.3598G>C	p.Ala1200Pro	De novo	Novel	
*SLC2A1*	1649	M	5.5	GTCS (4 y 8 m)	Mild	c.1477T>C	p.Ter493ArgextTer56	Mother	Novel	
*SYNGAP1*	5828	F	5	Atypical absences, Myoclonus, Atonic, GTCS, FS (10 m)	Severe	c.829dupC	p.Pro277ProfsTer7	De novo	Novel	
*UPF3B*	6189	M	2	IS (8 m)	Severe	c.883T>A	p.Leu295Met	Mother	Novel	
*ZEB2*	4620	M	3	PS (?)	Severe	c.1426_1427insA	p.Met476AsnfsTer6	De novo	Reported	

M, male; F, female; y, years; m, months; d, days; h, hours; PS, partial seizures; FS, febrile seizures; SE, status epilepticus; DS, Dravet syndrome; EIEE, early infantile epileptic encephalopathy; EE, epileptic encephalopathy; IS, infantile spasms; GS, generalized seizures; PDE, pyridoxine-dependent epilepsy; MMPSI, malignant migrating partial seizures in infancy; ESES, electrical status epilepticus in sleep; MICPCH, mental retardation and microcephaly with pontine and cerebellar hypoplasia

*have been reported in [12].

**Fig 1:**
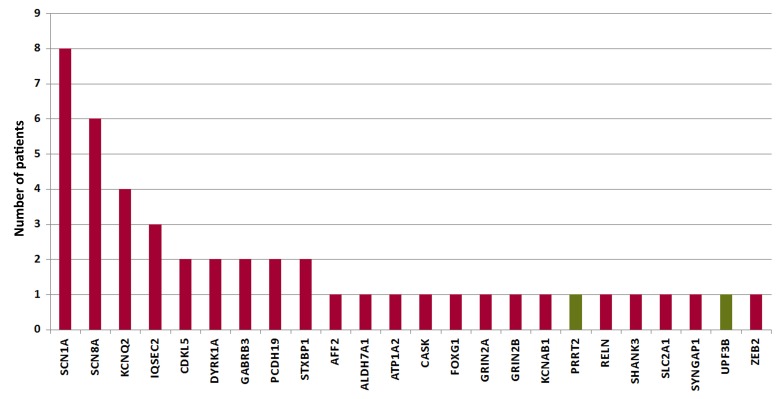
Pathogenic (red) and likely pathogenic (green) mutated genes identified in 253 patients with unexplained epilepsy and intellectual/developmental disabilities.

Among the 46 cases with a causative mutation, patients with an *SCN1A* mutation accounted for the largest proportion of 17% (8/46), followed by *SCN8A*, *KCNQ2* and *IQSEC2* of 13% (6/46), 9% (4/46) and 7% (3/46) respectively. A total of 38% (9/24) of the mutated genes (*SCN1A*, *SCN8A*, *KCNQ2*, *IQSEC2*, *CDKL5*, *DYRK1A*, *GABRB3*, *PCDH19*, and *STXBP1*) reoccurred at least two times in this study, and 63% (15/24) of the mutated genes (*ALDH7A1*, *AFF2*, *ATP1A2*, *CASK*, *FOXG1*, *GRIN2A*, *GRIN2B*, *KCNAB1*, *PRRT2*, *RELN*, *SHANK3*, *SLC2A1*, *SYNGAP1*, *UPF3B* and *ZEB2*) occurred only one time. All mutated genes detected in this study along with the number of patients in whom each gene was detected were shown in Fig 1.

Notably, we detected a novel *de novo* heterozygous mutation (c.1062dupCA p.Leu355HisfsTer5) within *KCNAB1* in one patient with EIEE. This mutation led to a premature termination codon. The patient with the mutation was a four-year-old girl with early-onset seizures (onset at ten days after birth). The frequent partial seizures were followed by intractable generalized tonic-clonic seizures (GTCS). Until now, she has used seven antiepileptic drugs (AEDs). Sodium Valproate, Levetiracetam, Topiramate and Clonazepam had no obvious effect, while Oxcarbazepine, Lamotrigine and Zonisamide reduced the seizures; among them Zonisamide add-on had the best effect with a 75% reduction of the seizures. Her parents are planning to consent for her to receive vagal nerve stimulation (VNS) to treat her intractable epilepsy. She also has severe ID/DD now (non-verbal, limited interaction with her parents, but can walk independent from 2 years 3 months), with occasional panic attacks. Her prenatal history was normal and neurological examination was unremarkable. One of her electroencephalograph (EEG) recordings showed a slow spike-wave in the left frontotemporal region, while another EEG recorded frequent sharp waves in the left temporooccipital region. Her cranial magnetic resonance imaging (MRI) was normal.

**Fig 2:**
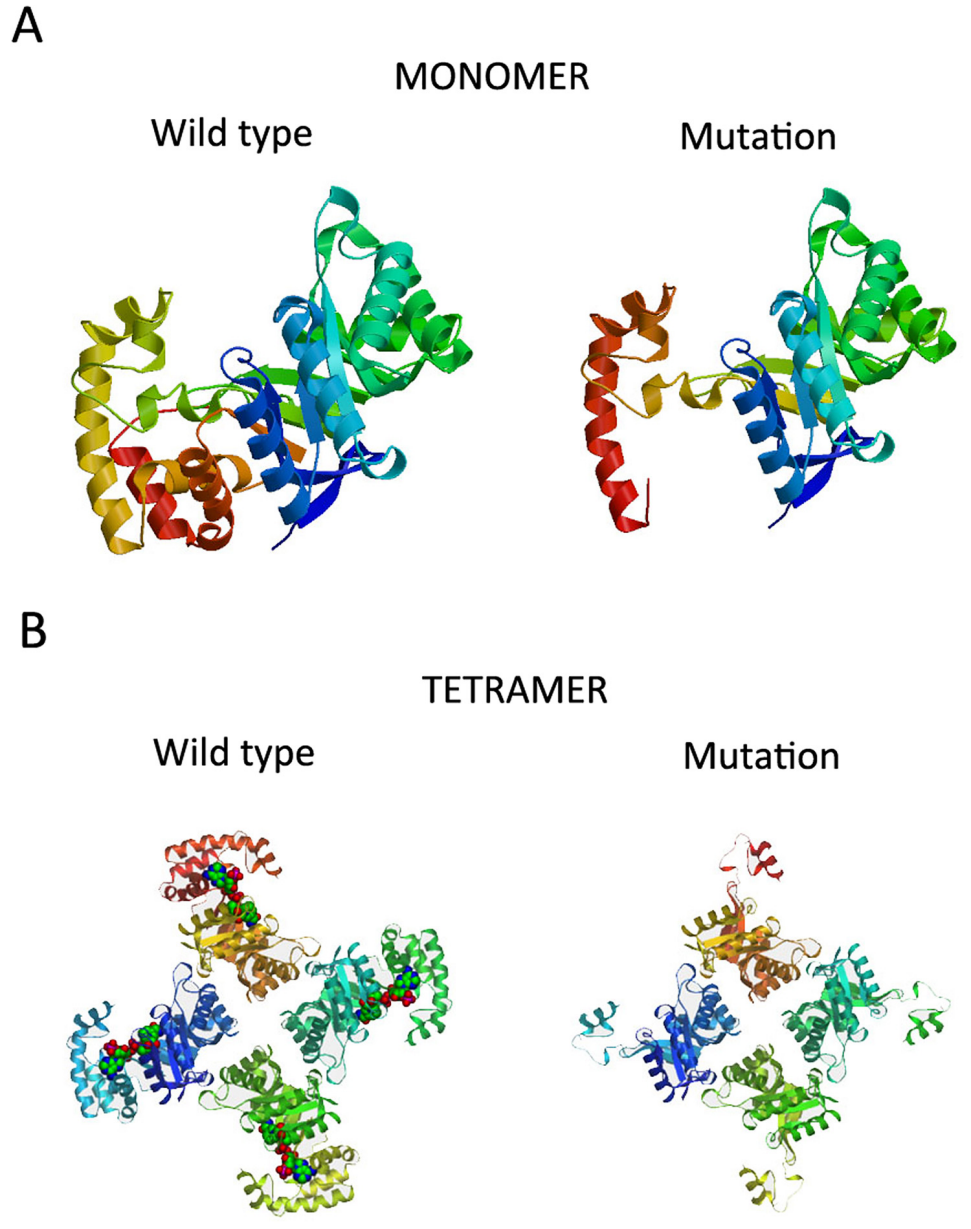
Structure modeling of wild type and p.Leu355HisfsTer5 mutation of *KCNAB1* with SWISS-MODEL. A: Monomer view: comparing with wild type, the mutant lost C-terminal structure of the protein; B: Tetramer view: comparing with wild type, the mutant might lose the ability of binding NADP^+^.

*KCNAB1* encodes the beta-1 member of the shaker-related family of voltage-gated potassium channels. This member includes three isoforms (Kvβ1.1- Kvβ1.3) of the *KCNAB1* gene [19–23]. Kvβ1.1, the longest isoform, is restricted expressed in brain [24]. The shaker-related voltage-gated K+ (Kv) channels consist of alpha and beta subunits [25]. The beta subunits modulate the gating properties of the alpha-subunit potassium channels. Voltage-dependent potassium channel proteins are responsible for the electrical properties of excitable cells and play physiological roles in non-excitable cells [26]. To further study the pathogenicity of p.Leu355HisfsTer5 mutation, protein tertiary structures of wild type and p.Leu355HisfsTer5 mutation of *KCNAB1* were predicted using the SWISS-MODEL. Because the template 3EAU was a homotetramers crystal structure, we predicted both monomer and tetramer of *KCNAB1* protein. According to the monomer prediction (Fig 2A), the mutated *KCNAB1* protein lost C-terminal helices when compared to wild type. According to the tetramer prediction (Fig 2B), the wild type was able to bind to NADP^+^ by sharing the same binding domains of *KCNAB2* protein (data from: http://www.uniprot.org/uniprot/Q14722). But the mutated *KCNAB1* protein was not able to bind to NADP^+^ for losing a NADP^+^ binding domain (375–381 amino acids), though might still be able to form a tetramer. Previous study of experimental point mutation within NADP^+^ binding domain of *KCNAB1* protein showed significant effects on Kv1 channel trafficking and axonal targeting [27]. Therefore, the p.Leu355HisfsTer5 mutation that we identified is probably pathogenic.

To understand a possible common genetic mechanism of epilepsy and ID/DD, we classified 24 mutated genes identified in our study into groups according to gene function. We found that ion channel genes had the largest percentage of occurrence, 33% (8/24) with genes related to synapse coming second at 21% (5/24). Other genes also identified were classified as having functions in transcriptional regulation, protein kinase modulation, cell metabolism and cell-cell interaction. The classification and the number of genes of each group were shown in Table 2.

**Table 2:** The classification of mutated genes.

Gene Function	Gene (n)	Mutated Genes
Ion channel	8	*SCN1A*, *SCN8A*, *KCNQ2*, *GABRB3*, *ATP1A2*, *GRIN2A*, *GRIN2B*, *KCNAB1*
Synapse	5	*IQSEC2*, *STXBP1*, *PRRT2*, *SHANK3*, *SYNGAP1*
Transcription regulation	4	*AFF2*, *FOXG1*, *UPF3B*, *ZEB2*
Protein kinase	3	*CDKL5*, *DYRK1A*, *CASK*
Cell metabolism	2	*ALDH7A1*, *SLC2A1*
Cell-cell interaction	2	*PCDH19*, *RELN*

## Discussion

In this study, we discovered 32 novel and 16 reported mutations within 24 genes in 46 patients of our cohort, including two likely pathogenic mutated genes in two patients. The total detection rate of our study was 18% (46/253) in the whole group and 26% (17/65) in the early-onset epilepsy group. Early-onset epilepsy had the relatively higher detection rate. We made genetic diagnosis for these 46 patients. This was critical for them to improve further management and genetic counseling for their epilepsy. We expanded the phenotype and mutation spectrum of the 24 genes identified in our study. This provided more information for further understanding of these disease-causing genes. Patients with an *SCN1A* mutation accounted for the largest proportion, 17% (8/46), of which seven patients were diagnosed as Dravet syndrome and one patient was diagnosed as MMPSI. MMPSI cases with a *SCN1A* mutation have been reported and MMPSI is regarded as the most severe phenotype of *SCN1A* to date [28, 29]. Our MMPSI patient with a novel *SCN1A* mutation provided further evidence that *SCN1A* defects play an important role in MMPSI. A total of 63% (15/24) of the mutated genes occurred only one time in our study; therefore, it seems that epilepsy and ID/DD are phenotypes that occur as a consequence of brain dysfunction caused by highly diverse mutated genes, most of which are isolated and fit the rule of common disease rare variations. In addition, the spectrum of mutated genes in our study is rather different from those reported in other similar studies [11, 30]. Population diversity and different inclusion criteria (both for patients and candidate genes) may account for this inconsistency.

In this study, we identified a novel *de novo* heterozygous mutation (c.1062dupCA p.Leu355HisfsTer5) within *KCNAB1* in one patient with EIEE. *KCNAB1* has been reported as a susceptibility gene for epilepsy, particularly temporal lobe epilepsy (TLE), but no pathogenic mutation has been reported. An association study of 2717 epileptic patients reported that numerous SNPs located within *KCNAB1* contributed to the susceptibility to epilepsy. These patients manifested various forms of epilepsy [31]. Furthermore, *KCNAB1* was regarded as a candidate gene for lateral temporal epilepsy (LTE) because of its functional interaction with *LGI1* [32], the disease-causing gene of autosomal dominant LTE (ADLTE). However, sequencing of *KCNAB1* in ADLTE families without *LGI1* mutations failed to identify any mutations. This suggested that *KCNAB1* does not act as a major disease-causing gene in ADLTE [33]. Nevertheless, another association study of 142 LTE patients suggested that *KCNAB1* may be a susceptibility gene of LTE [34]. In addition; a genome-wide scan study was conducted on a TLE family. Linkage analysis identified a locus on chromosome 3q25-q26. *KCNAB1* was one of the highest priority genes in this region, but sequencing of *KCNAB1* was unable to identify any mutations [35]. In summary, although several previous studies have supported the association of *KCNAB1* with epilepsy, no *KCNAB1* mutations have been reported in patients with this disease previously. However, a mouse model of *KCNAB1* disruption showed significant alterations in hippocampal learning and memory functions [36], supporting a possible relationship between *KCNAB1* defects and brain dysfunction. Here we reported an epileptic patient with a *KCNAB1* mutation, which supports the relationship between *KCNAB1* dysfunction and epilepsy, and interestingly, the epileptic discharges of this patient located mostly at temporal region.

We also found four patients with a *KCNQ2* mutation. Because *KCNQ2* [37] and *KCNT1* [38] have already been reported to be common and important genes for epileptic encephalopathy, adding the recently reported *KCNA2* [39], *KCNH1* [40], *KCNC1* [41] genes and now the *KCNAB1* gene, this led us to pay more attention to the potassium channel genes as a group in epilepsy, especially epileptic encephalopathy.

Two mutated genes were regarded as likely having pathogenic mutation in our study. First, we detected a heterozygous mutation (p.Arg217ProfsTer8) within *PRRT2* in one family (5240). The proband had severe ID/DD and infantile spasms, but other individuals with the same mutation in this family had benign epilepsy during infancy and normal intelligence during adulthood. We hypothesized that the *PRRT2* mutation in the proband may only increase the risk of epilepsy, while another undiscovered mutated gene may instead contribute to the severe phenotype. In addition, we discovered a hemizygous mutation (p.Leu295Met) in *UPF3B* which have been reported as a causative gene of X-linked recessive mental retardation. The previously reported cases with mutation in *UPF3B* had no seizures. The mutation we found is novel, but probably damaging predicted by Polyphen2. Unfortunately, DNA samples of other male maternal family members were unavailable to make sure the pathogenicity of this mutation.

To understand a possible common genetic mechanism of epilepsy and ID/DD, we classified the mutated genes identified in our cohort according to gene function. We have found that ion channel genes had the largest percentage of occurrence. This suggests that ion channels play a vital role in the pathogenesis of epilepsy and ID/DD. Activation of neurotransmitter receptor ion channels at synapses promotes synaptic plasticity during brain development. Therefore, abnormal ion transport may affect neural excitability and brain development, resulting in epilepsy and ID/DD [42]. Further, synapse formation and normal function are essential in the signaling and the formation of neural networks. Genes related to synapse formation and function were also closely related to epilepsy and ID/DD. In addition, some factors in transcriptional regulation, protein kinase modulation, cell metabolism and cell-cell interaction may also participate in the common pathogenesis of epilepsy and ID/DD. However, relevant details remain unclear. We believe further study of the common pathogenesis of epilepsy and ID/DD are urgently needed.

In our study, with the detection rate of 18%, the targeted NGS is certainly supposed to be an efficient and precise approach to screen monogenic mutations in patients with highly heterogeneous disorders such as epilepsy and ID/DD. However, according to our experience, some limitations of this approach and tips for best performance should be discussed here. First, owing to false positive results, conventional Sanger sequencing is definitely required for the validation of the variations supposed to be significant, especially when the targeted regions have insufficient coverage. Second, on the other hand, false negative results may also occur and may lead to the loss of crucial data. This might be one of the reasons that our study failed to detect gene mutations in the other 207 patients. Third, DNA samples of the parents and other affected or even unaffected members of families are essential to analyze the pathogenicity of the variations. Availability of almost all parental DNA samples in our study played a significant role in data analysis. However, the unavailability of other members in a few families hampered further confirmation of their etiology. Finally, precise clinical data is a prerequisite, without which the genetic diagnosis cannot be made. For example, patient 5871 who carried a *de novo* mutation in *ATP1A2* also had an inherited homozygous mutation (p.Ile105Val) in *CLN3*. Although the *CLN3* nonsynonymous mutation was predicted to be “probably damaging”, we still excluded its pathogenicity according to his phenotype, not like neuronal ceroid lipofuscinoses clinically.

In summary, we used targeted NGS to investigate causative gene mutations in Chinese children with unexplained epilepsy and ID/DD. We established genetic diagnosis for 46 patients of our cohort and expanded the phenotype and mutation spectrum of 24 genes associated with epilepsy and ID/DD. This study is the first to identify a *KCNAB1* mutation in a patient with EIEE. More cases with mutations in this gene are needed to confirm and clarify its role in epilepsy.
